# Single irrigation at the four-leaf stage in the spring optimizes winter wheat water consumption characteristics and water use efficiency

**DOI:** 10.1038/s41598-022-18446-8

**Published:** 2022-08-22

**Authors:** Xiaoyuan Bao, Xuejing Liu, Xiaoyang Hou, Baozhong Yin, Weiwei Duan, Yandong Wang, Jianhong Ren, Limin Gu, Wenchao Zhen

**Affiliations:** 1 College of Agronomy, Hebei Agricultural University, Baoding, China; 2 State Key Laboratory of North China Crop Improvement and Regulation, Baoding, 0710001 People’s Republic of China

**Keywords:** Biological techniques, Biotechnology, Plant sciences

## Abstract

Water scarcity is a key constraint to crop production in North China Plain (NCP), which produces the majority of the country’s winter wheat (*Triticum aestivum* L.). The objective of this three-year field study was to see whether and when irrigation one-time in spring improved grain productivity and water use efficiency. Four sets of irrigation were established at the 3-leaf visible stage (L3) and the L4, L5, and L6 stages. When irrigation time was postponed, the spike number, 1000-grain weight, and water consumption increased progressively, whereas grain yield, grain number, dry matter, harvest, and WUE grew, then dropped, and peaked at L4. The increased grain number can be attributed to the L4's higher daily water consumption and water consumption percentage throughout the jointing-anthesis stages compared to the L3, L5, and L6. The cumulative (37 days), whereas it was longer in L3, L5, and L6(40, 42, and 43 days, respectively). Furthermore, flag leaf senescence was postponed in L4 with a higher post-anthesis leaf area index, photosynthetic rate, chlorophyll content, higher superoxide dismutase activity, and lower malondialdehyde concentration. As a result, single irrigation at the 4-leaf visible stage optimized water deficit and consumption before and after anthesis, resulting in higher yield and WUE in the NCP.

## Introduction

Wheat (*Triticum aestivum* L.) is the most widely planted and important cereal grain in the world. In regions where fresh water is scarce, wheat production is restricted. One of the most effective ways to relieve the conflict between food demand and water shortage was the adoption of optical irrigation management^1^.

The global per capita water supply is around 9000 m^3^, but only 2200 m^3^ in China. While it is 456 m^3^ in North China Plain (NCP), one of China's important grain-producing regions, which accounts for about 60% of the country’s wheat output^2,3^. Climate change, rising food demand, and water limitations have become a major challenge to the sustainable development of agriculture in the NCP^4^. Wheat requires 400–500 mm of water, but the typical rainfall in this region is only 50–150 mm, which is insufficient and results in unpredictable wheat growth^5,6^. Thus, irrigation is the primary way for enhancing the growth and development of winter wheat and achieving a higher yield^7^. The region’s primary source of irrigation is groundwater, which is applied to winter wheat through surface irrigation^8^. Winter wheat is traditionally irrigated three to six times with a total volume exceeding 300 mm, which significantly reduces the water use efficiency (WUE) of winter wheat^9–11^. The groundwater in the North China Plain is severely overdrawn, generating the world's largest groundwater funnel area^12^. In this context, crop production has shifted from high-yield style to water-saving strategies^13^. The current water-saving and high-efficiency irrigation scheme in this region now irrigated twice at the jointing stage and anthesis period^14,15^. However, the problem of groundwater overexploitation remains serious. 13 × 10^4^ ha field was fallowed to prohibit groundwater irrigation and mitigate the declining trend of groundwater levels. Winter wheat may be transplanted the fallowe area if the irrigation volume was reduced further. Therefore, in order to prevent further declines in the grougdwater table, the NCP urgently needs to reduce irrigation while maintaining productivity to further improve the WUE^16,17^.

Optimizing the irrigation schedule is an important measure to improve the winter wheat yield and WUE^18–20^. Limited irrigation is usually defined as an irrigation strategy in which less irrigation water is consumed lower than the total required by the crops, i.e., reducing the number of irrigation times and irrigation amount, which can be considered to save water and increase the WUE^21,22^. For surface irrigation, reducing the irrigation times is more realistic, especially when labor is scarce.

There has been a substantial amount of research on single irrigation in the spring, with varying outcomes; nonetheless, it has been demonstrated that, in general, single irrigation reduces or maintains the yield while increasing the WUE in comparison to conventional irrigation^23,24^. Previous studies on single irrigation in spring were based primarily on the growth stage, but there have been few studies on irrigation based on the leaf age of wheat in the spring^19^. In comparison to growth stages, leaf age was also significantly related to nutritive organs and process of spike differentiation. The wheat leaf age model is an easily observable exterior morphological index that is not only accurate, specific, and obvious, but also simple to understand and applicable from wheat sowing to maturity. However, the optimal leaf age for single irrigation remained unknown and required further research. Drought stress induces a series of physiological and biochemical responses in plants, and flag leaf senescence is directly dependent on the intensity of drought stress^25,26^. A moderate delay in irrigation time can improve the yield, harvest index (HI) and WUE by promoting the growth of wheat roots into deeper soil and increasing the water absorption of deeper soil^27^. Moderate stress promoted dry matter transport to the kernel, while mild stress at the recovery-jointing stage improved the structure of wheat canopy before anthesis and sustained higher post-anthesis photosynthesis^28^. Controlling soil moisture prior to jointing stage not only increases wheat root growth but also controls the leaf size and tiller number, therefore decreasing the needless use of water and fertilizer^5^. The flag leaf is the last senescent leaf and is regarded as the primary contributor during the grain filling stage^29^. The chlorophyll content, photosynthetic rate, and antioxidant enzyme activity could reflect the severity of drought stress in winter wheat^30^. Reactive oxygen species (ROS) created by drought stress can cause the peroxidation of lipids in cell membranes. Superoxide dismutase(SOD) is an essential component of the antioxidant protection enzyme system, which can reduce the cytotoxic effects of ROS^31^. Malondialdehyde (MDA) is a byproduct of membrane lipid peroxidation that indicates crop cells damage under unfavorable conditions^32^. During grain filling, water stress causes a reduction in photosynthesis and acceleration of leaf senescence, which are principally responsible for the decline in wheat yield^33^.

Based on the spring leaf age of winter wheat, the effect of single irrigation on wheat water consumption and yield was investigated in this was performed based on the. It was hypothesized that irrigation at a certain leaf age could optimize the supply of soil water before and after the anthesis of wheat, which not only ensures the formation of spike numbers and grain numbers but also delays post-anthesis leaf senescence and increases the dry matter accumulation during grain filling. To test this hypothesis, a three-year experiment with vary irrigation periods was conducted, and the leaf area index and dry matter accumulation, post-anthesis leaf photosynthetic rate and chlorophyll content, the activity of SOD, the content of MDA, yield and yield components, and the characteristics of water consumption were investigated.

## Materials and methods

### Experimental site description

**Figure 1:**
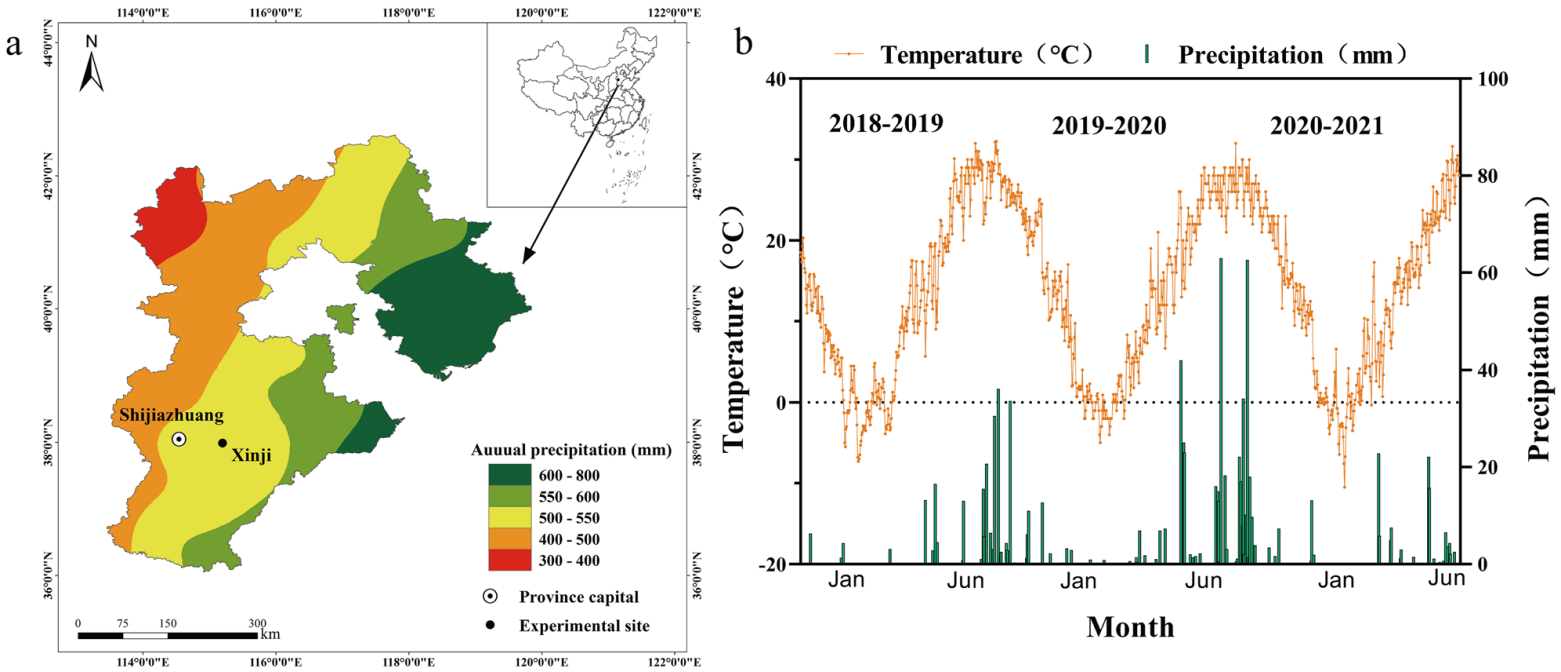
The location of experimental field (**a**) and as well as rainfall and temperature during the three growthing seasons (**b**).

The field experiments were conducted during the 2018–2021 growing seasons at the Malan Experimental Station of the Agricultural University of Hebei, Baoding, China (37°99′N, 115°20′E). The region is located in the semi-humid continental temperate monsoon region with an altitude of 37 m. In the topsoil (0–40 cm) of the testing field, the concentrations of organic matter, total nitrogen, available potassium, available phosphorus and pH were 22.4 g kg^−2^, 1.26 g kg^−2^, 125.0 mg kg^−2^, 25.8 mg kg^−2^ and 7.6 respectively. We have counted the precipitation in Hebei Province in recent 20 years, and the test site is shown in (Fig. 1a) The average annual rainfall from 2001 to 2021 was 472.5 mm, including 124.5 mm in the wheat season (from October 10 to June 10). Daily rainfall and daily mean temperature for the three growth seasons of winter wheat were shown in Fig. 1b. The rainfall during the three winter wheat seasons was 85.9 mm, 176.2 mm and 103.4 mm, respectively.

### Experimental design

The experiment adopted a randomized block experiment design. The soil bulk density and field capacity of the 0–200 cm soil layers in 20 cm increments are shown in Table 1. The high-yielding wheat cultivar Shi4366 was utilized, as it is one of the most important and widely cultivated crops in Hebei Province. The planting density was 3.75 × 10^4^ plants ha^−1^ with a row spacing of 15 cm.

**Table 1:** The soil bulk density and field capacity in 0–200 cm soil layers in the experimental plots.

Soil layer (cm)	0–20	20–40	40–60	60–80	80–100	100–120	120–140	140–160	160–180	180–200
Bulk density (g cm^−3^)	1.34	1.59	1.55	1.60	1.60	1.59	1.60	1.60	1.59	1.58
Field capacity (%)	35.2	35.3	37.6	37.7	38.0	35.9	35.0	35.0	33.2	34.6

**Figure 2:**
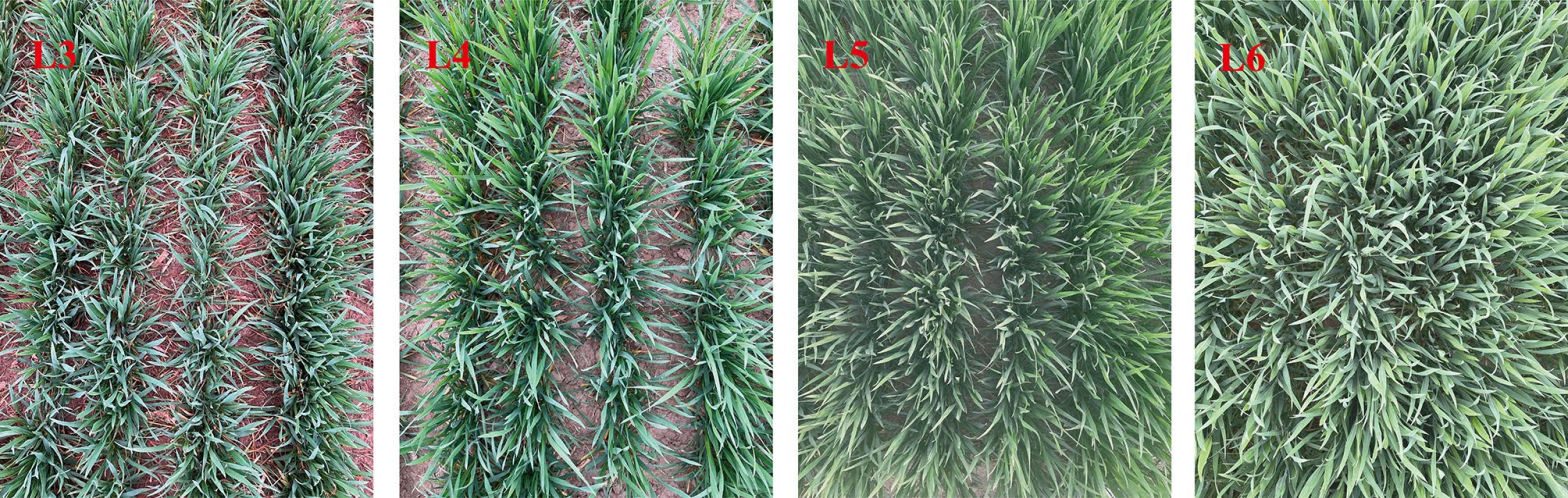
The winter wheat growth condition of the four irrigation timesWheat field growth before spring irrigation.

The wheat was sown on October 10, 2018, October 13, 2019, and October 10, 2020, and the harvest dates were June 5–8, 2019, June 4–7, 2020, and June 5–7, 2021, respectively. Four irrigation time at the 3-leaf visible (L3) stage, 4-leaf visible (L4) stage, 5-leaf visible (L5) stage, and 6-leaf visible (L6) stage were tested with 90 mm irrigation level. The growth of wheat in the field during the irrigation period is shown in Fig. 2. The irrigation method was micro-spray irrigation, with a spraying height of 0.6 m and a spraying range of 1.2 m. The base fertilizer, including 120 kg N ha^−1^, 112.5 kg P_2_O_5_ ha^−1^ and 112.5 kg K_2_O ha^−1^, was applied with a rotary cultivator before sowing. A rate of 120 kg N ha^−1^ of urea (46% N) was completely dissolved in a fertilization device and applied as topdressing together with the irrigation water. Each treatment was repeated three times in three replicates for a total of 15 plots. Each test plot was composed of 60 rows of wheat. The row spacing was 15 cm. Each treatment was conducted in three test plots with an area of 20 m^2^. The dates of sowing, the jointing stage, anthesis stage, harvest stage and irrigation date for the three seasons are listed in detail in Table 2.

**Table 2:** Dates of sowing, jointing stage, anthesis stage, and harvest under different treatments in the 2018–2021 growing seasons of winter wheat.

Year	Treatment	Dates of sowing	Dates of jointing stage	Dates of anthesis stage	Dates of harvest	Irrigation date
2018–2019	L3	October 8, 2018	April 5, 2019	May 4, 2019	June 6, 2019	March 25, 2019
	L4	October 8, 2018	April 3, 2019	May 2, 2019	June 8, 2019	April 3, 2019
	L5	October 8, 2018	April 3, 2019	May 2, 2019	June 7, 2019	April 11, 2019
	L6	October 8, 2018	April 3, 2019	May 1, 2019	June 5, 2019	April 17, 2019
2019–2020	L3	October 13, 2019	April 3, 2020	May 2, 2020	June 6, 2020	March 22, 2020
	L4	October 13, 2019	April 1, 2020	April 30, 2020	June 7, 2020	March 30, 2020
	L5	October 13, 2019	April 1, 2020	April 30, 2020	June 7, 2020	April 8, 2020
	L6	October 13, 2019	April 1, 2020	April 29, 2020	June 4, 2020	April 15, 2020
2020–2021	L3	October 10, 2020	April 5, 2021	May 4, 2021	June 5, 2021	March 24, 2021
	L4	October 10, 2020	April 5, 2021	May 2, 2021	June 7, 2021	April 3, 2021
	L5	October 10, 2020	April 5, 2021	May 2, 2021	June 7, 2021	April 11, 2021
	L6	October 10, 2020	April 5, 2021	May 1, 2021	June 5, 2021	April 17, 2021

### Sampling and measurements

#### Soil water

The content of volumetric water of each 20 cm soil layer to a depth of 0–200 cm (Trime Pico 64 Portable Soil Moisture Meter, TDR, EMIKO, GmbH, Bochum, Germany) was monitored at the sowing stage, returning green stage, jointing stage, anthesis stage and every five days from the 3-leaf unfolding stage to maturity. After the first irrigation, we set three stress levels, which are mild stress (soil relative water content 65–70%); moderate stress (soil relative water content 55–65%); Severe stress (soil relative water content < 55%)^34^. The monitoring depth is 0–60 cm. The water consumption (ET) was calculated using the following equation:

1 $${{\rm E}{\rm T}}_{1-2}=\Delta S+{\rm M}+{\rm P}{\rm O}+{\rm K}$$

2 $$PO=\alpha P$$

where ${{\rm E}{\rm T}}_{1-2}$ is the phase water consumption (mm); $\Delta S$ is the change in the soil water storage in the 2 m soil body during the wheat growth period (mm) (i.e., the soil storage water consumption); ${\rm M}$ is the irrigation amount (mm) for the period; ${\rm P}{\rm O}$ is the effective precipitation (mm), P is single precipitation (mm) and α is effective utilization coefficient precipitation (when the precipitation is less than 50 mm, α = 1.0, when the precipitation is 50-150 mm, α = 0.80, when the rainfall is more than 150 mm, α = 0.7). ${\rm K}$ is the groundwater replenishment amount (mm) for the period. The groundwater depth in the experimental site is lower than 25.8 m, and the K value can be ignored.

The water consumption percentage for a given stage (CP) is the ratio of the water consumption amount of that stage (CA) to the total evapotranspiration (ET). The daily water consumption (CD) is the ratio of the number of growing days at this stage to the water consumption amount of that stage.

where Y is the grain yield (kg ha^−1^).

The water use efficiency (WUE, kg m^3^) was calculated as follows:

3 $$WU{\rm E}=\Upsilon/{\rm E}{\rm T}$$

### Dynamics of winter population

Tillers were marked from the appearance of the first tiller. The newly initiated tillers of each plant were checked and tagged every 5 days. After jointing, the tagged plants were selected and separated based on the tiller positions for measuring. In this study, the main stem was considered to be O, and the primary tillers that grew from the true leaf axillary of O were considered to be I, II, III, and IV among others. Conversely, I-p, I-1, and I-2 among others were used for the secondary tillers that grew from the axillary of the true leaf of the primary tillers^35,36^.

The number of tillers (stems) per square meter was investigated at the wintering stage, returning green stage, jointing stage, heading stage, anthesis stage, and maturity.

### Leaf area index (LAI) and Dry matter accumulation (DM)

The green leaf area was measured every 5 days from the anthesis stage to maturity (i.e., 0, 5, 15, 20, 25, and 30 days after anthesis) using an Li-3100 area meter (LICOR, Inc., Lincoln, NE, USA), and the green leaf area index (LAI) was calculated.

To determine the dry matter accumulation, plants with an area of 0.15 m^2^ at ground level were sampled at anthesis and maturity, respectively, and all the plant samples were oven-dried at 105 ℃ 30 min, and then oven-dried at 75 °C to a constant weight. The HI was calculated as the ratio of grain yield (GY) to the total aboveground DM accumulated at maturity.

### Leaf chlorophyll content and photosynthetic rate

To determine the chlorophyll content (a and b) of the flag leaf from anthesis to maturity, 20 flag leaf samples were randomly collected from each experimental plot every 5 days (i.e., 0, 5, 15, 20, 25, and 30 days after anthesis) and stored at − 25 °C until the biochemical assays were performed. The leaf samples were chopped on ice, quickly weighed (200 mg), and extracted with 95% ethanol for 48 h in the dark. The extract was measured at A649 and A665 with a spectrophotometer.

The wheat flag leaves were sampled at 0, 5,15, 20, 25, and 30 d after the anthesis stage. The photosynthetic rates (Pn, μmol CO_2_ m^−2^ s^−1^) were measured using a Li-6800 Portable Photosynthesis System (LI-COR Biosciences).

### Antioxidant enzyme activities and the content of malondialdehyde

Flag leaves were collected at 10:00 every 10 days from anthesis. The flag leaves were ground in 5 mL of extraction buffer (50 mM potassium phosphate + 0.4% polyvinylpyrrolidone [PVP], pH 7.0) on dry ice. The extract was centrifuged at 10,000 g for 15 min at 4 °C. The supernatant was collected to measure the content of MDA and activity of SOD as described by^37^.

### Grain yield and yield components

To determine the final grain yield, the spikes were counted in six 1 m central rows of each plot before harvest. The number of grains per spike was determined by calculating the number of grains per spike on 50 randomly selected plants from each plot. The weight of 1000 grains (TGW, 13% water content) was calculated by weighing 1000 grains three times in each sample. During the physiological maturity of wheat, 3 m^2^ of wheat plants were harvested and threshed from each plot to determine the grain yield (13% moisture content).

### Statistical analysis

Statistical analyses were performed using SPSS 19.0 (SPSS, IBM, USA). The effects of the treatment were investigated using a typical split-plot design analysis method. Significant differences were identified using a one-way analysis of variance (ANOVA) and least significant difference (LSD) tests at 95% or 99% confidence levels. All the figures were created using ArcGIS 10.2 or GraphPad Prism 9.0 (GraphPad Software, Inc., San Diego, CA, USA, https://www.graphpad.com/). A correlation analysis was performed using R software (R 4.0.4, https://www.r-project.org/).

## Results

### Grain yield, yield components and WUE

Grain yield, spike number, grain number and 1000-grain weight (TGW) of winter wheat were significantly affected by the year (Y) and irrigation time(I) (Table 3). The grain yield and TGW were also significantly affected by the interaction of year and irrigation time. The spike number increased initially and then decreased with the delay in the irrigation time, and peaked in the L4 treatment. Compared with L4, the average spike number in L3, L5 and L6 were reduced by 4.6%, 9.9% and 17.2%, respectively. However, the grain number decreased with the delay of irrigation time, showing that L4, L5, and L6 were 3.9%,7.8% and 16.5% lower than L3, respectively. The grain number in L6 was significantly lower than that in the other treatments, while the difference between L3, L4 and L5 was not significant. However, the TGW significantly increased with the delay of irrigation time. Compared with L3, the average TGW increased by 1.9 g, 2.4 g, and 2.9 g with each leaf age delay in irrigation.

**Table 3:** Effects of irrigation time on grain yield, yield components and water use efficiency in winter wheat. Different letters indicate significant difference in treatments at *P* < 0.05 level. Y, year; I, irrigation time. *** indicate significant effects at *P* < 0.001, respectively; ns indicates no significant effect. Values are means ± standard error (*n* = 3). ANOVA, analysis of variance; WUE, water use efficiency.

Year	Treatment	Spike number (× 10^4^ ha^−1^)	Grain numbers per spike	1000-grain weight(g)	Grain yield (kg ha^−1^)	WUE (kg ha^-1^ mm^−1^)
2018–2019	L3	700.7 b	34.3 a	30.5 d	6692.7 b	16.5
	L4	740.3 a	33.1 a	32.7 c	7644.2 a	19.5 a
	L5	676.5 b	31.5 ab	34.1 b	6601.6 b	17.3 b
	L6	594.0 c	28.8 b	37.9 a	5884.0 c	16.1 c
2019–2020	L3	779.9 ab	29.3 a	35.5 d	7436.9 ab	16.6 b
	L4	818.4 a	27.5 a	37.5 c	7631.3 a	17.6 a
	L5	726.0 bc	26.3 ab	41.1 b	7259.3 b	17.0 b
	L6	676.5 c	23.3 b	43.6 a	6012.9 c	14.4 c
2020–2021	L3	674.3 ab	35.3 a	34.7 d	7626.6 b	17.6 b
	L4	699.6 a	34.4 ab	36.2 c	8233.3 a	19.5 a
	L5	632.5 bc	33.4 ab	38.3 b	7555.4 b	18.1 b
	L6	600.6 c	30.5 b	40.7 a	6518.8 c	16.5 c
ANOVA	Year (Y)	***	***	***	***	***
	Irrigation time(I)	***	***	***	***	***
	Y × I	ns	ns	***	***	***

Compared with 2020–2021, the grain yield of 2018–2019 and 2019–2020 was significantly reduced. The variation in annual grain yield was attributed to rainfall and other meteorological factors. The lower precipitation after anthesis in 2018–2019 resulted in a lower TGW, and the low temperature in the spring of 2019–2020 resulted in a lower spike number (April 9–10). Despite the annual variation in GY, the L4 treatment produced the highest yield over the three growth seasons. Other treatments had significantly lower GY than L4, except in 2019–2020 when there was no significant difference in the GY of L3 and L4. In comparision to L4, the average GY of L3, L5 and L6 decreased by 7.5%, 8.9% and 21.7%, respectively.

### Leaf area index (LAI), dry matter accumulation and harvest index (HI)

**Figure 3:**
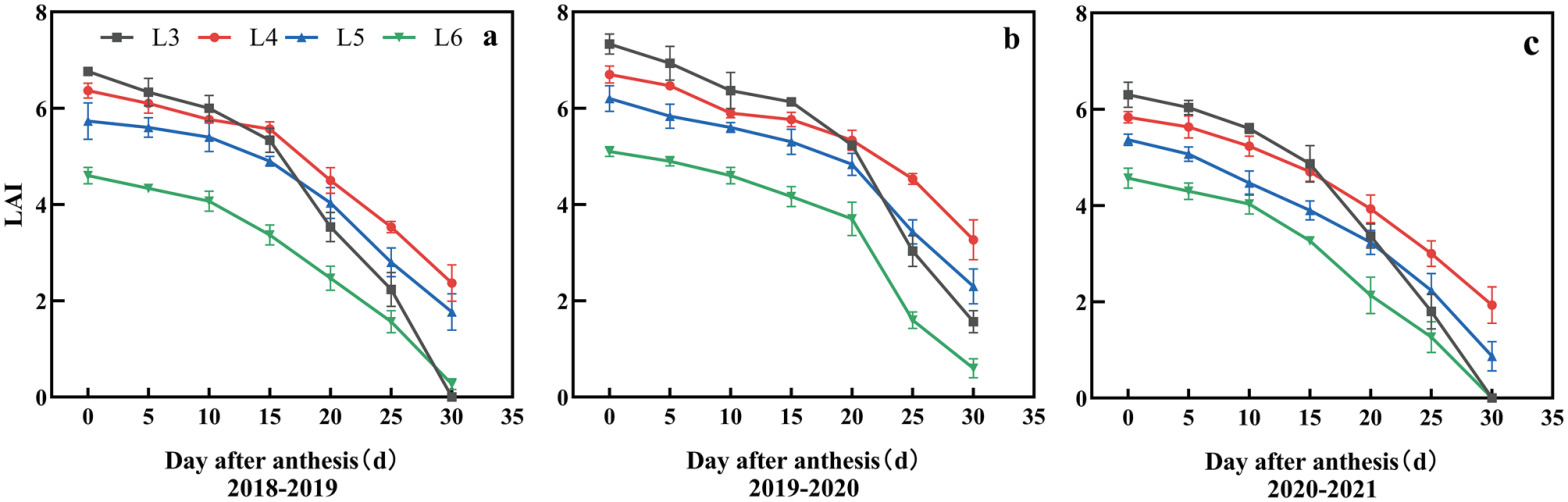
Effects of irrigation time on leaf area index after flowering anthesis stage in 2018–2019 (**a**), 2019–2020 (**b**) and 2020–2021 (**c**).

The maximum LAI at the anthesis stage was recorded at L3, and the three-year average was 6.8. As irrigation time was delayed from L3 to L4, L5 and L6, the LAI decreased by 7.4%, 15.2% and 30.1%, respectively. (Fig. 3). After the anthesis period, the LAI of each treatment began to decrease, but the range of decrease differed. L3 and L6 decreased significantly from 15 days after anthesis (DAA) in 2018–2019 and 2020–2021, while those from 15 DAA responded similarly in 2019–2020. The LAI of L4 and L5 decreased from DAA 15–20, but the rate of decrease was lower than those of L3 and L6. After 20 DAA, the LAI of L4 and L5 were significantly higher than those of the other treatments. This indicated that a moderate delay in irrigation time could effectively prolong the LAI of grain filling in the middle and late stages.

**Figure 4:**
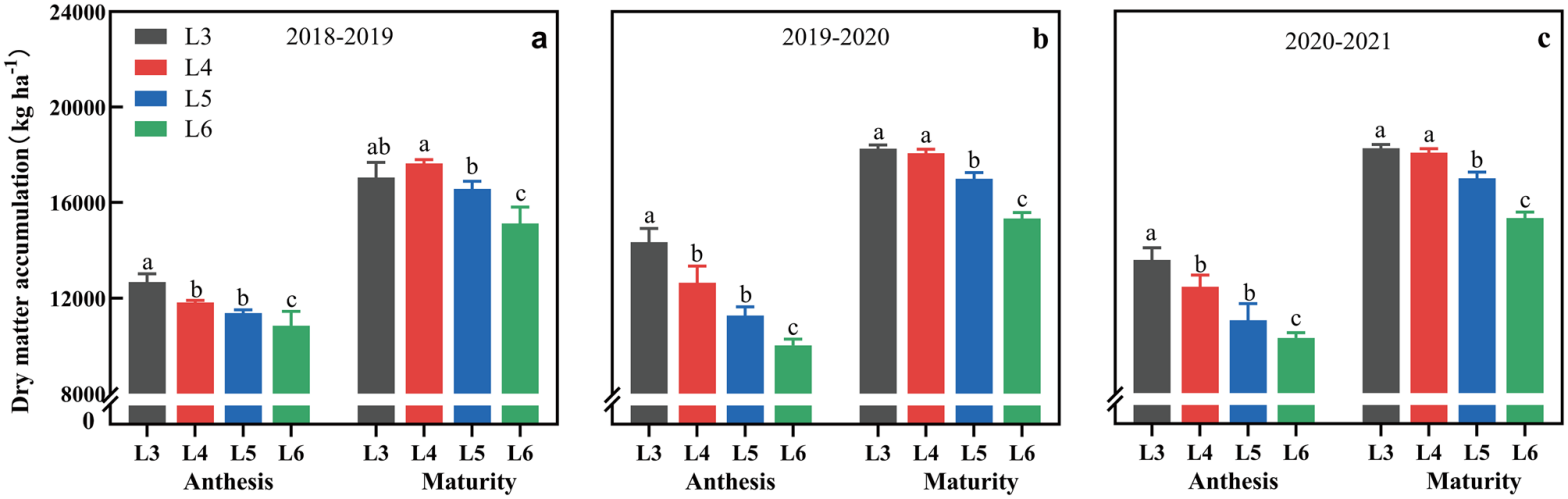
Effects of different irrigation treatments time on dry matter accumulation (DMA) at flowering and DMM at maturity in 2018–2019 (**a**), 2019–2020 (**b**) and 2020–2021(**c**).

Dry matter accumulation at the anthesis stage (DMA) decreased with the delay of irrigation time (Fig. 4), and the DMA at L3 was significantly higher than those of the other treatments. Dry matter accumulation at maturity (DMM) reached its maximum value at L4, with a three-year average of 17,925.7 kg ha^−1^, and there was no significant difference between L3 and L4. However, they were significantly higher than those of L5 and L6. The DMM of L5 and L6 were 6.0% and 14.9% lower than that of L4, respectively.

**Figure 5:**
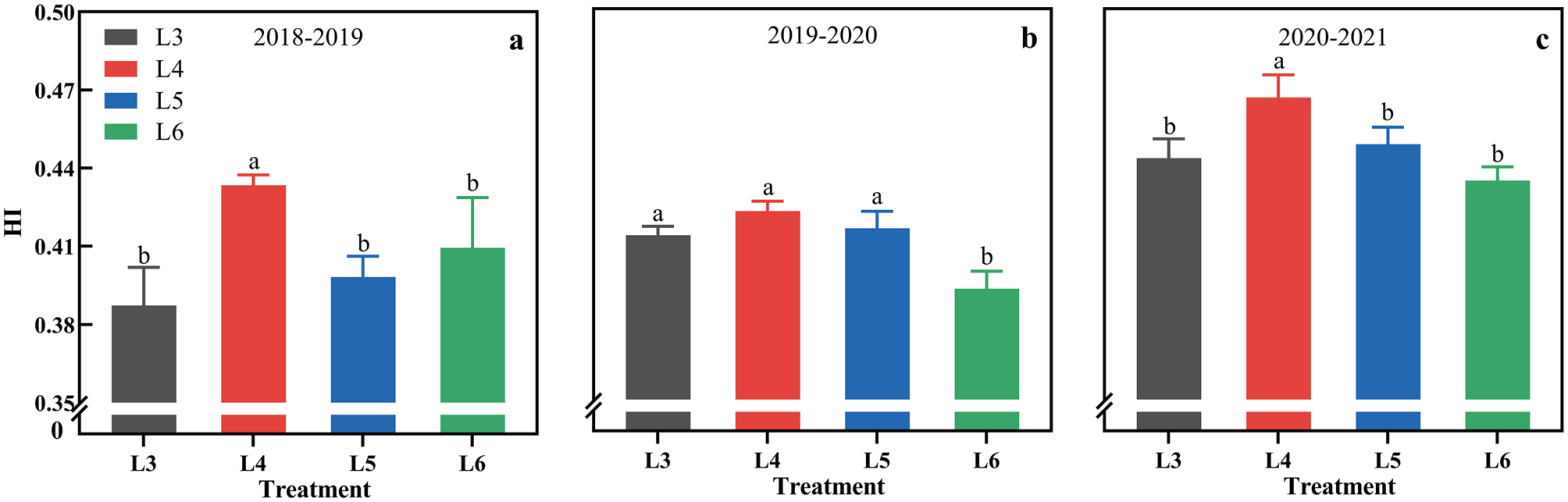
Effects of different irrigation treatments time on harvest index in 2018–2019 (**a**), 2019–2020 (**b**) and 2020–2021 (**c**).

There were differences in the HI values during the three years owing to the effects of the DMA and grain yield. However, L4 achieved the highest HI over three years with an average of 0.44 (Fig. 5), which was significantly higher than that of other treatments in 2018–2019 and 2021–2022. Compared with L4, L3, L5 and L6 reduced the HI by 6.0%, 4.5% and 6.5%, respectively.

### Water consumption

**Figure 6:**
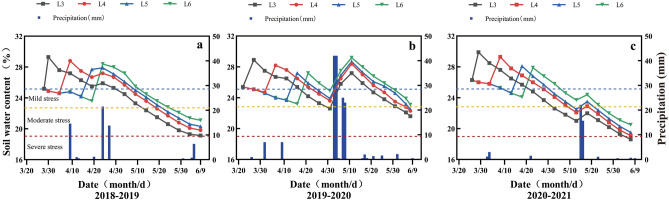
Average water content of the 0–60 cm layer of farmland soil under different treatments in 2018–2019 (**a**), 2019–2020 (**b**) and 2020–2021 (**c**) and precipitation and irrigation in the test area. According to the Mild stress (soil relative water content 65–70%); Moderateild stress (soil relative water content 55–65%); Severe stress (soil relative water content < 55%).

Before the first irrigation in spring, the soil moisture in L4, L5, and L6 all reached the level of mild stress (Fig. 6). The duration of drought exposure increased with the delay of irrigation time, showing an average of 5, 13, and 18 days in L4, L5, and L6, respectively. On the anthesis stage, the soil moisture in L3 reached the moderate drought stress level in 2021–2022, while other treatments reached the mild drought stress level. The duration of post-anthesis drought stress gradually decreased with the delay in irrigation time, and there were differences in the influence of rainfall in different years. From the 3-leaf age irrigation to maturity, the plants in L4 were exposed to drought stress for the shortest duration (37 d on average) during the three-year period, while L3, L5, and L6 were 40 d, 42 d, and 43 d, respectively.

The proportion of irrigation water in the total water consumption was 20.3%, 20.1% and 21.6% on average during three wheat growing seasons in 2019, 2020, and 2021, respectively. Precipitation accounted for 22.3%, 39.2% and 24.8% of the total water consumption during these years, respectively. The proportion of soil water consumption to total water consumption was 54.4%, 36.2% and 53.7% on average during these years, respectively (Table 4). This shows that the precipitation ratio only slightly varied under different annual types, while precipitation as a percentage of ET and soil water consumption as a percentage of ET varied substantially under different annual types, which was caused by different amounts of rainfall during the wheat season.

**Table 4:** Effects of irrigation time on total water consumption (ET), soil water consumption (ΔSW) and their water consumption ratios during the 2018–2021 wheat growing seasons. *, **, and *** indicate significant effects at *P* < 0.05, *P* < 0.01 and *P* < 0.001.

Year	Treatment	ET (mm)	I (mm)	P (mm)	ΔSW (mm)	Ratio to total water consumption (%)		
						Irrigation	Precipitation	Soil water
2018–2019	L3	405.4 a	90	85.9	229.5 a	22.2 c	21.2 c	56.6 a
	L4	391.6 b	90	85.9	215.7 b	22.9 b	21.9 b	55.1 b
	L5	381.8 b	90	85.9	205.9 b	23.6 b	22.5 b	53.9 b
	L6	366.0 c	90	85.9	190.1 c	24.6 a	23.5 a	51.9 c
2019–2020	L3	446.8 a	90	176.2	180.7 a	18.2 c	35.5 c	36.4 b
	L4	432.3 b	90	176.2	166.1 b	20.1 b	39.4 b	38.2 a
	L5	427.6 b	90	176.2	161.5 bc	20.8 a	40.8 b	37.4 a
	L6	417.2 c	90	176.2	139.6 c	21.1 a	41.2 a	32.6 c
2020–2021	L3	434.3 a	90	103.4	240.9 a	20.7 b	23.8 b	55.5 a
	L4	423.1a	90	103.4	229.7 a	21.3 b	24.5 b	54.3 a
	L5	416.5 ab	90	103.4	223.1 ab	21.6 ab	24.8 ab	53.8 ab
	L6	396.0 b	90	103.4	202.6 b	22.7 a	26.1 a	51.2 b
ANOVA	(Y)	***			***	***	***	***
	(I)	***			***	***	***	***
	Y × I	**			***	**	***	*

Year (Y), irrigation (I) treatment and their interaction significantly affected the total water consumption and stage water consumption. With the delay in the time of irrigation, the water consumption and soil water consumption gradually decreased. Compared with L3, delaying the irrigation time (L4, L5, and L6) reduced the consumption of water by 13.2 mm, 20.2 mm and 35.8 mm for L4, L5 and L6, respectively (Table 4). In 2018–2020, there was no significant difference in the consumption of total water between L4 and L5, but they were significantly lower than L3 but higher than L6. In 2020–2021, the difference in total water consumption between L3, L4 and L5 was not significant, but they were significantly higher than that of L6. The total soil water consumption in the three growth seasons was consistent with the trend of rainfall, which is highest in 2019–2020, followed by 2020–2021 and 2018–2019. This was a result of increased rainfall-induced increases in soil consumption and evapotranspiration.

Year and irrigation significantly had a significant influence on water consumption (CA), water consumption intensity (CD), and water consumption modulus coefficient (CP) in various growth periods, and the interaction of year and irrigation had a sign influence on CA (Table 5). From the sowing to jointing stages, the CA, CD and CP of irrigated treatment (L3)were significantly higher than those of the non-irrigated treatments (L4, L5, and L6). From the jointing to anthesis stages, CA and CD were significantly higher in L3, L4 and L5 than in L6, while CP was L4 > L5 > L3 > L6. From anthesis to maturity, CA in L3 was significantly lower than those of L4, L5, and L6. The results showed that winter wheat can use more water during the grain-filling period when irrigation is delayed.

**Table 5:** Water consumption characteristics in different wheat growth stages.

Year	Treatment	Sowing to jointing			Jointing to anthesis			Anthesis to maturity		
		CA (mm)	CD (mm d^−1^)	CP (%)	CA (mm)	CD (mm d^−1^)	CP (%)	CA (mm)	CD (mm d^−1^)	CP (%)
2018–2019	L3	175.4 a	1.0 a	43.3 a	115.3 a	4.0 a	28.7 ab	114.7 b	3.5 b	28.7 c
	L4	145.4 b	0.8 b	37.0 b	119.3 a	4.1 a	30.7 ab	126.9 a	3.4 b	32.3 b
	L5	145.4 b	0.8 b	38.0 b	112.3 a	3.9 a	29.3 a	124.1 a	3.4 b	32.7 b
	L6	145.4 b	0.8 b	39.7 b	90.3 b	3.2 b	24.7 b	130.3 a	3.7 a	35.7 a
2019–2020	L3	173 a	1.0 a	38.7 a	115.0 a	4.0 a	25.7 bc	158.8 b	4.5 b	35.7 c
	L4	140.8 b	0.8 b	32.7 b	125.9 a	4.3 a	29.3 a	165.6 ab	4.4 b	38.3 bc
	L5	140.8 b	0.8 b	33.0 b	119.5 a	4.1 a	28.0 ab	167.3 ab	4.4 b	39.3 ab
	L6	140.8 b	0.8 b	34.0 b	99.5 b	3.5 b	24.0 c	176.9 a	4.9 a	42.3 a
2020–2021	L3	189.4 a	1.0 a	43.7 a	123.6 ab	4.3 ab	28.7 ab	121.4 b	3.8 b	27.7 c
	L4	152.8 b	0.9 b	36.3 b	130.8 a	4.5 a	31.0 a	139.5 a	3.9 b	33.0 b
	L5	152.8 b	0.9 b	36.7 b	123.4 ab	4.3 ab	29.7 a	140.3 a	3.9 b	33.7 b
	L6	152.8 b	0.9 b	38.3 b	101.4 b	3.4 b	24.0 b	141.8 a	4.2 a	37 a
ANOVA	Year(Y)	***	ns	***	***	ns	ns	***	***	***
	Irrigation(I)	***	***	***	***	***	***	***	***	***
	Y × I	ns	ns	ns	*	ns	ns	**	ns	ns

CA, water consumption amount; CD, daily water consumption; CP, water consumption percentage; I, irrigation; Y, year.

### Dynamics of the wheat population

**Figure 7:**
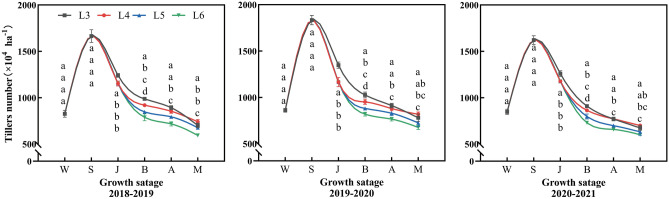
Dynamics of winter wheat tills number (× 107 ha-1) at different growth stages in 2018–2019 (**a**), 2019–2020 (**b**) and 2020–2021 (**c**). W, S, J, A, B and M indicate the growth stages of wintering, standing, jointing, booting, anthesis, and maturity, respectively. Each value represents the mean ± SE (n = 3). Bars showing the same letter are not significantly different at P ≤ 0.05 as determined by the LSD test.

The tiller number of wheat in different treatments showed a unimodal change, peaking at the standing stage, and then declining with the growth process (Fig. 7). At standing stage, the total tiller number in the irrigated treatment (L3) was significantly higher than those in the non-irrigated treatments (L4, L5, and L6). From jointing to anthesis stage, the tiller death rate of L3, L4 and L5 increased with the delay in irrigation time. Compared to L3, the average number of tillers at booting stage decreased by 6.4%, 13.6%, and 20.0%, while at anthesis stage it decreased by 2.7%, 9.8% and 16.8%, respectively. The tiller number in L3 decreased rapidly than L4, L5 and L6 after anthesis, resulting in a lower spike number than L4, with the difference reaching statistical significance in 2018–2019 (Fig. 7a). These results indicated that delayed irrigation reduced the number of tillers before anthesis, whereas early irrigation (L3) led a sharp reduction in tiller numbers after anthesis.

### The senescence process and photosynthetic characteristics

**Figure 8:**
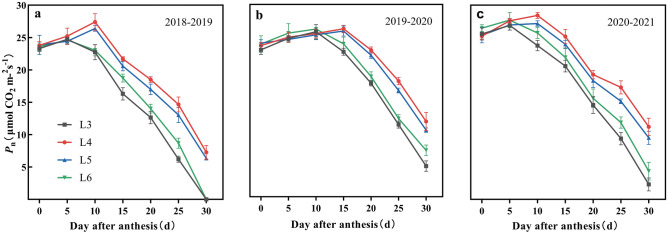
Effects of irrigation time on photosynthetic rate of flag leaves in 2018–2019 (**a**), 2019–2020 (**b**) and 2020–2021 (**c**) years. Vertical bars represent standard errors. Pn, synthetic rate.

**Figure 9:**
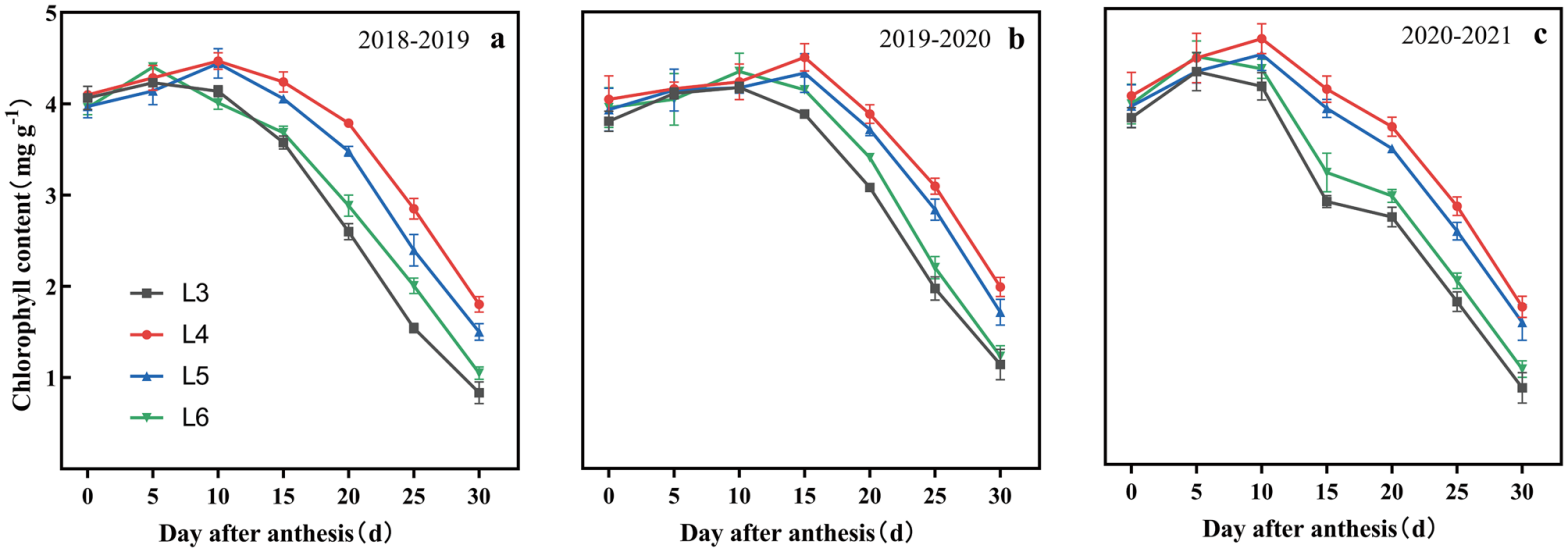
Effects of irrigation time on chlorophyll content of flag leaves in 2018–2019 (**a**), 2019–2020 (**b**) and 2020–2021 (**c**). Vertical bars represent standard errors.

The photosynthetic rate (Pn) and chlorophyll content (Chl) showed a consistent trend of first increasing and then decreasing after anthesis, but the peak time differed among treatments (Figs. 8 and 9). L3 and L6 reached their peaks at 5–10 DAA, while L4 and L5 reached their peaks at 10–15 DAA. Both Pn and Chl obtained their maximum values at L4, which were 27.3 μmol CO_2_ m^−2^ s^−1^ and 4.5 mg g^−1^, respectively. The Pn peaks of L3, L5, and L6 were 5.8%, 3.2%, and 4.4% lower than those of L4, respectively. In addition, the Chl peaks of L3, L5, and L6 were 6.8%, 2.7%, and 3.0% lower than those of L4. This indicated that moderately delayed irrigation in L4 increased the maximum photosynthetic rate and chlorophyll content. The Pn and Chl trends after the peak were L4 > L5 > L6 > L3, and the Pn and Chl of L3 and L6 decreased rapidly compared with those of L4 and L5.

**Figure 10:**
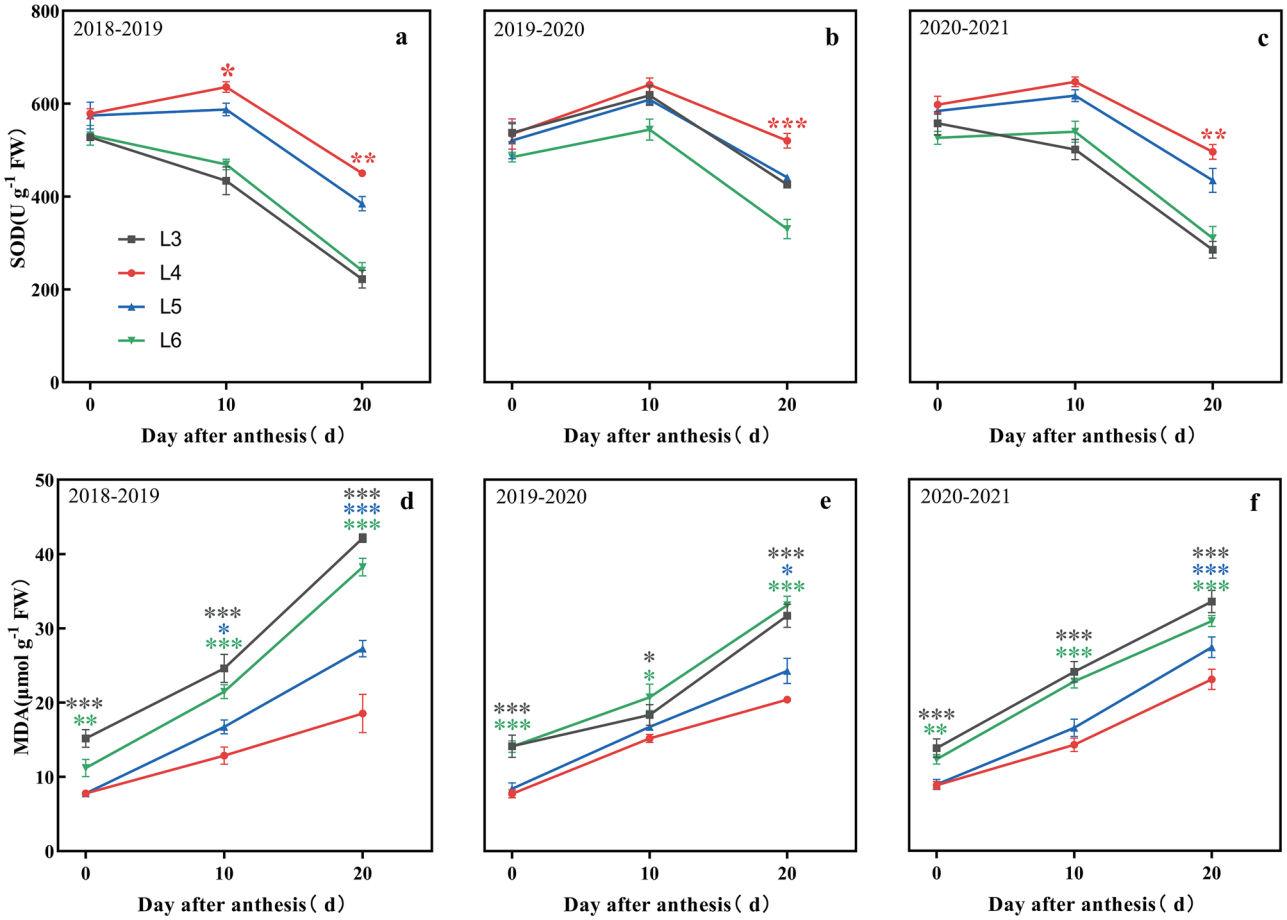
Effects of irrigation time on antioxidant system of flag leaf. SOD, superoxide dismutase; MDA, malondialdehyde. *, ** and *** indicate significance at the 0.05, 0.01 and 0.001 levels, respectively.

Compared to other treatments, L4 significantly increased the SOD activity in flag leaves after anthesis (Fig. 10a–c). The accumulation of MDA in L3 and L6 at the anthesis stage was significantly higher than that in L4, and the difference gradually increased with the progress of the filling period (Fig. 10d–f). The accumulation of MDA in L5 was significantly higher than that of L4 at 10 to 20 DAA. Overall, L4 enhanced the activity of leaf antioxidant enzyme activity and inhibited the excessive accumulation of MDA, which ultimately delayed leaf senescence.

### Correlation analysis of yield factors, water consumption characteristics and physiological indicators

**Figure 11:**
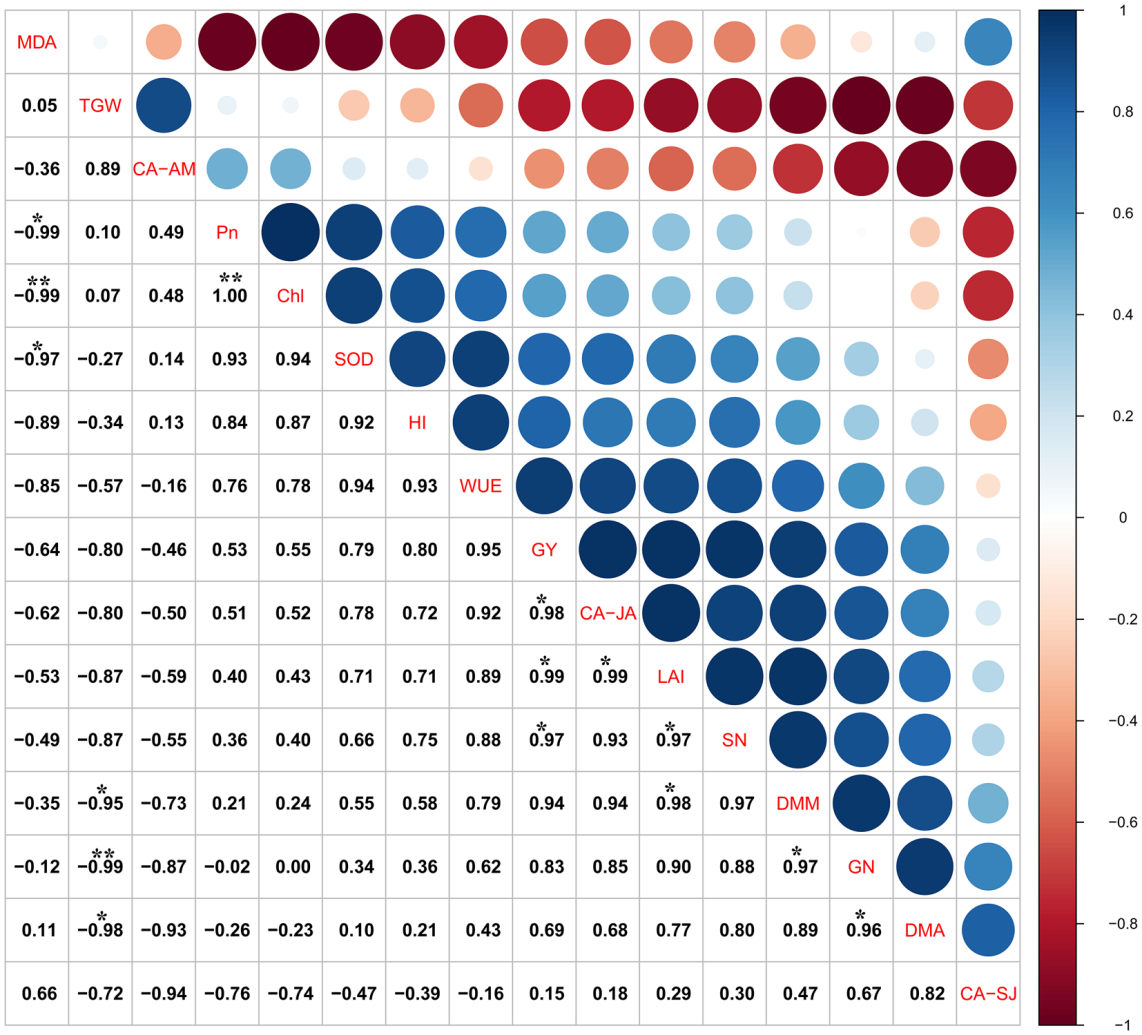
Spearman correlation coefficient matrix and 95% confidence interval of each morphological index is indicated as follows: *P < 0.05; **P < 0.01. GY, grain yield; SN, spike number; GN, grain number; TGW, 1,000-grain weight; WUE, water use efficiency; CA-SJ, water consumption from snowing to jointing; CA-JA, water consumption from jointing to anthesis; CA-JA, water consumption from anthesis to maturity; DMA, dry matter accumulation at anthesis; DMM, dry matter accumulation at maturity; LAI, leaf area index; HI, harvest index; Pn, photosynthetic rate; Chl, Chlorophyll; MDA, malondialdehyde; SOD, superoxide dismutase.

A significant positive correlation was observed between GY, SN and LAI, whereas LAI significantly positively correlated with DMM and water consumption from jointing to anthesis (CA-JA)(Fig. 11). CA-JA also significantly positively correlated with GY. GN significantly positively correlated with DMM and MDA. There was a significant positive correlation between Pn and Chl. TGW significantly negatively correlated with DMM, GN and DMA. MDA significantly negatively correlated with Pn, Chl and SOD. No significant correlation was observed between HI, WUE, and the other indicators.

## Discussion

Limiting crop irrigation during non-critical growth stages is an advanced irrigation management strategy. This irrigation approach cannot fully meet the needs of crops, but it is required to precisely manage the watering time^38,39^. Previous studies have shown that the limited irrigation technique can reduce water consumption and improve WUE^40,41^. Currently, water-saving cultivation is a promising technology in the region. Accurate division of crop growth stage is the basis of managing water and fertilizer reasonably to improve crop water and fertilizer utilization efficiency^42^. Leaf-age irrigation was an irrigation strategy using the number of leaves on the main stem in the spring to divide the winter wheat or rice growth stages and precisely regulate the wheat’s growth and yield formation^43^.

In this study, under the premise of sufficient soil moisture before sowing, winter wheat was irrigated once with different spring leaf ages as irrigation periods. The three-year experimental results showed that the yield and WUE under limited water irrigation varied between 6012.9 kg ha^−1^ and 8233.3 kg ha^−1^, 14.4 kg mm^−1^and 19.6 kg mm^−1^, respectively (Table 3). The treatment with single irrigation at the spring 4-leaf age in the spring achieved a higher grain yield and WUE. This indicated that under the condition of single irrigation in spring in the North China Plain, irrigation at the 4-leaf age of wheat in spring was the optimal time for water use efficiency and grain yield.

### Effects of irrigation on water use efficiency

Irrigation time is an important factor for a single irrigation^43^. The three-year results showed that delayed irrigation reduced the water consumption of winter wheat by up to 19.7% compared with L3, while the WUE reached its maximum in L4. L3 was subjected to drought stress for the longest time after anthesis, which resulted in the early senescence of flag leaves and a reduction in the 1000-grain weight. L5 and L6 were subjected to mild drought stress for a longer time before irrigation, during the critical phase of panicle development and stem-tiller differentiation, resulting in different degrees of reduction in panicle number and grain number per panicle. From the beginning of irrigation to the maturity stage, the drought stress time of L4 was the shortest compared with the other treatments, resulting in a better soil environment before and after the anthesis of wheat (Fig. 6). Simultaneously, L4 showed the highest CA, CD and CP during the jointing-anthesis stage, which also indicated that the utilization of water at this stage was ultimately beneficial to the formation of yield (Table 5). CA, CD and CP from the anthesis stage to maturity stage were the highest in L6, but L6 did not produce higher yields, which could be owing to a large amount of ineffective water evaporation caused by the smaller population.

### Effects of grain yield and yield components

The key growth stage of winter wheat is the jointing and grain filling stages, during which water shortages can lead to severe decreases in grain yield. However, some studies have demonstrated that limited water irrigation in the specific growing period of winter wheat does not result in a decrease in yield and can even maintain or even increase yields^45^. There is a mutually beneficial relationship between WUE and yield, and in areas < 200 mm of precipitation during the growing season and loamy or sandy soil, deficit irrigation can effectively improve the WUE^46^. Guo et al^29^reported that irrigation one time increased the WUE and soil water consumption, and the WUE decreased with the increase in irrigation. The yield of winter wheat is closely related to aboveground biomass production and HI^45^. One-time irrigation at jointing increased the ratio of contribution of dry matter after anthesis to grain, thus, improved the HI and GY^47^. Some studies have shown that irrigation can enhance the LAI, delay leaf senescence, and increase the duration of leaf area^48^. Limited irrigation between the jointing and anthesis stages significantly increased the wheat yield and WUE by increasing both current photosynthesis and the remobilization of pre-anthesis carbon reserves^49^. In this study, delaying irrigation to the 4-leaf age (i.e., L4) did not significantly reduce the number of grains but slightly increased the spike numbers. It significantly increased the 1,000-grain weight (Table 3), which was the reason for the higher yield at L4. Although higher grain weights were obtained when the plants were irrigated at the 5- or 6-leaf ages (i.e., treatments L5 and L6), this treatment eventually resulted in significantly lower yields owing to a significant reduction in the numbers of ears and grains per ear (Table 3). This shows that early or late irrigation will not help to obtain a higher yield, and there is no significant difference between L3 and L4 in 2019–2020, which could be owing to more precipitation in April and May (Fig. 1b). For 3-leaf-age irrigation, highest LAI and DMA were obtained at the anthesis stage. This could be related to the fact that the irrigation at the 3-leaf age in spring resulted in the vigorous growth of wheat populations pre-anthesis. However, a reduction in post-anthesis LAI and leaf senescence were delayed by postponing irrigation to the 4-leaf age, which promoted the DMA at the grain-filling stage and increased the contribution of post-anthesis dry matter to the grain, which, in turn, increased yield. This is consistent with previous studies that found that moderately low growth in the LAI of wheat does not result in low yields and moderate water deficits in March can save water^47,50^.

### Effects of irrigation on Chl, Pn and LAI

Previous research showed that the physiology and growth of wheat are affected by soil water content^39^^.^ Muhammad^51^ found that winter wheat is extremely tolerant to mild and moderate abiotic stress before jointing, and properly delaying irrigation can delay leaf senescence, increase the Pn, increase the activity of SOD, and reduce the concentration of MDA.

In this study, the changes in SOD activity and MDA concentration differed among various treatments in the three wheat growing seasons (Fig. 10). After 10 DAA, the SOD activity in L4 maintain high, which negatively correlated with the content of MDA. At 20 DAA, the activity of SOD in L4 was significantly higher than that of the other treatments, while the concentration of MDA was significantly lower than that of the other irrigation treatments. The results showed that different irrigation time with same irrigation amount had variable effects on the post-anthesis antioxidant capacity of winter wheat. The activity of SOD can be effectively activated in the L4 treatment, which inhibited the accumulation of MDA. This was consistent with the trend of variation of Pn and Chl in flag leaves. This is consistent with the findings of previous studies^52^.

## Conclusions

Under limited water conditions, leaf age irrigation is a subtractive and effective irrigation management strategy that could achieve higher yield and WUE with one-time irrigation at the critical stage, when the crops are most sensitive to water. This study evaluated the effects of different irrigation time on the GY, WUE and physiological characteristics of the flag leaves of winter wheat based on a three-year field experiment. In comparison to other treatments, 4-leaf-age irrigation increased the water consumption during jointing to anthesis, resulting in a higher grain number. 4-leaf-age irrigation shorted the accumulative duration of drought stress, delayed leaf senescence, enhanced the activity of SOD, reduced the concentration of MDA, and increased LAI, Pn and dry matter accumulation. While 5-leaf age irrigation and 6-leaf age irrigation exacerbated drought stress before irrigation, and significantly reduced LAI and antioxidant properties, resulting in premature senescence of flag leaves and lower grain number. Therefore, one-time irrigation at the 4-leaf stage in the spring balanced the drought stress before and after irrigation, and was the best irrigation scheme to save water and generate high yields of winter wheat in the North China Plain.

## Data Availability

The datasets used and/or analyzed during the current study are available from the corresponding author on reasonable request.
